# Education Attainment, Intelligence and COVID-19: A Mendelian Randomization Study

**DOI:** 10.3390/jcm10214870

**Published:** 2021-10-22

**Authors:** Gloria Hoi-Yee Li, Stanley Kam-Ki Lam, Ian Chi-Kei Wong, Jody Kwok-Pui Chu, Ching-Lung Cheung

**Affiliations:** 1 Department of Health Technology and Informatics, The Hong Kong Polytechnic University, Hong Kong, China; gloria-hy.li@polyu.edu.hk; 2 School of Nursing, Li Ka Shing Faculty of Medicine, The University of Hong Kong, Hong Kong, China; stanleylam@cuhk.edu.hk; 3 The Nethersole School of Nursing, Faculty of Medicine, The Chinese University of Hong Kong, Hong Kong, China; 4 Department of Pharmacology and Pharmacy, Li Ka Shing Faculty of Medicine, The University of Hong Kong, Hong Kong, China; wongick@hku.hk (I.C.-K.W.); chukpj@hku.hk (J.K.-P.C.); 5 Research Department of Practice and Policy, School of Pharmacy, University College London, London WC1E 6BT, UK

**Keywords:** education, intelligence, SARS-CoV-2, COVID-19, Mendelian randomization

## Abstract

Background: Evidence of socioeconomic inequality in COVID-19-related outcomes is emerging, with a higher risk of infection and mortality observed among individuals with lower education attainment. We aimed to evaluate the potential interventions against COVID-19 from the socioeconomic perspective, including improvement in education and intelligence. Methods: With a two-sample Mendelian randomization approach using summary statistics from the largest genome-wide association meta-analysis, univariable analysis was adopted to evaluate the total causal effects of genetically determined education attainment and intelligence on COVID-19 outcomes. Multivariable analysis was performed to dissect the potential mechanisms. Results: Genetic predisposition to higher education attainment by 1 SD (4.2 years) was independently associated with reduced risk of COVID-19 severity (OR = 0.508 [95% CI: 0.417–0.617]; *p* < 0.001). Genetically higher education attainment also lowered the risk of COVID-19 hospitalization (0.685 [0.593–0.791]; *p* < 0.001), but the association was attenuated after adjustment for beta estimates of intelligence in multivariable analysis. Genetically higher intelligence was associated with reduced risk of COVID-19 hospitalization (0.780 [0.655–0.930]; *p* = 0.006), with attenuation of association after adjustment for education attainment. Null association was observed for genetically determined education attainment and intelligence with SARS-CoV-2 infection. Conclusion: Education may act independently and jointly with intelligence in improving the COVID-19 outcomes. Improving education may potentially alleviate the COVID-19-related health inequality.

## 1. Introduction

A novel severe acute respiratory syndrome coronavirus 2 (SARS-CoV-2) was identified to cause a cluster of pneumonia cases in Wuhan, China, in December 2019 [1]. The World Health Organization (WHO) has characterized the coronavirus disease (COVID-19) as a pandemic on 11 March 2020. As of 1 June 2021, the global number of confirmed cases of COVID-19 and related deaths has reached 170.4 and 3.5 million, respectively.

Evidence of socioeconomic inequalities emerge in the incidence and mortality of both non-communicable and infectious diseases, including COVID-19 [2]. Socioeconomic status (SES) is affected by social determinants such as education, income and ethnicity, which are also the key social determinants of health. Notably, the mortality from infectious diseases in individuals with elementary or lower education level was approximately two-fold of individuals with higher education level [3]. Lower education level was reported to be associated with a stronger agreement with COVID-19 misinformation [4], poorer knowledge and practices regarding COVID-19 protection [5,6], which may, in turn, lead to increased risk of SARS-CoV-2 infection or worse COVID-19 outcomes. Although studies have reported that lower education attainment was associated with increased risk of SARS-CoV-2 infection, COVID-19 hospitalization or mortality [7,8,9,10,11,12,13], whether the relationship is causal remains unclear. Notably, education attainment is phenotypically and genetically correlated with intelligence, and bidirectional causation exists between the two traits [14]. Yet, investigation of their independent association with the risk of SARS-CoV-2 infection or COVID-19 outcomes would have different implications in devising policies combating COVID-19. If lower education attainment elevates the risk of SARS-CoV-2 infection or COVID-19 outcomes independently of intelligence, or education is on the causal pathway from intelligence to COVID-19, implementation of policy to lengthen the years of schooling might likely lower the COVID-19 related risk. Alternatively, if intelligence affects the risk of COVID-19 outcomes independently of education attainment, or intelligence mediates the education-COVID-19 association, provision of adequate training to improve the cognitive functions might be more effective in combating COVID-19. Meanwhile, a very recent population-based case-control study in Scotland showed that teachers were subjected to a reduced risk of COVID-19 related hospital admission and severe COVID-19 when compared to the general population [15]. We hypothesize that their higher education attainment or intelligence may be the causes for much lower risk. In this study, we firstly adopted univariable Mendelian randomization (MR) approach to investigate the total causal effects of education attainment and intelligence on SARS-CoV-2 infection, COVID-19 hospitalization and severity. In case causal association exists, multivariable MR was performed to examine the presence of potential mediators in the causal pathway.

## 2. Materials and Methods

### 2.1. Study Design and Data Sources

**Figure 1:**
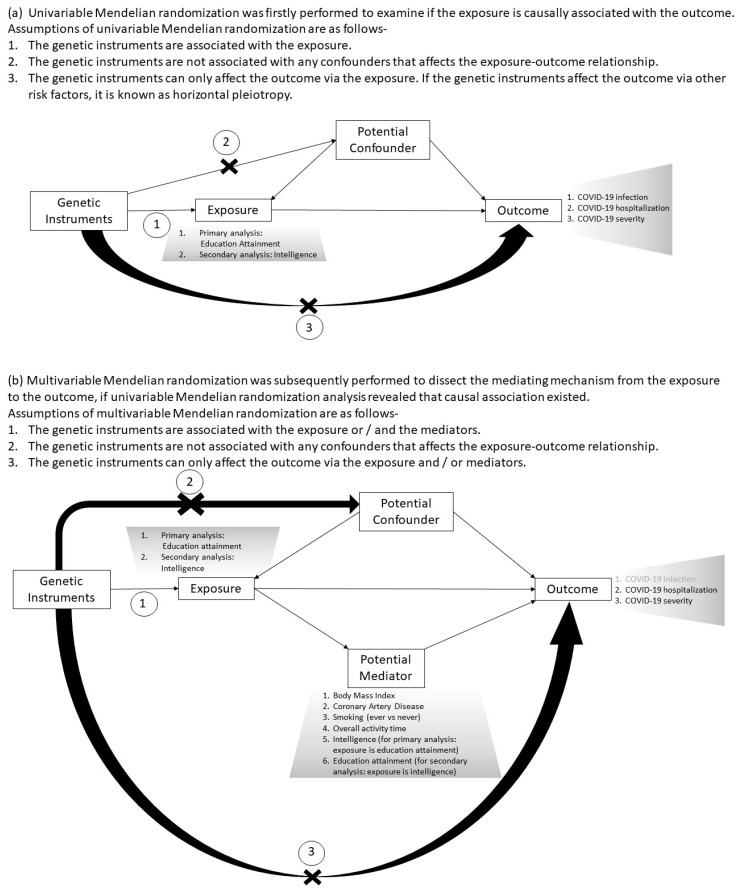
Study design and key assumptions in Mendelian randomization analyses. (**a**) Assumptions of univariable Mendelian randomization. (**b**) Assumptions of multivariable Mendelian randomization.

The study design is illustrated in Figure 1. In this two-sample MR study, we firstly examined if the exposures are causally associated with the COVID-19 outcomes by univariable MR analysis. The largest publicly available genome-wide association studies (GWAS) or GWAS meta-analysis of exposure (education attainment [16] (defined as the number of years of schooling; N = 1,131,881 individuals from 71 cohorts) and intelligence [17] (a latent factor denoted as general intelligence or Spearman’s *g*; N = 269,867 individuals from 14 cohorts)) and outcome (SARS-CoV-2 infection, COVID-19 related hospitalization and severity [18]) were used as the data sources. All these studies had obtained informed consent from participants and ethics approval from the respective institutional review board. In January 2021, the COVID-19 Host Genetics Initiative (COVID-19 HGI) released the latest meta-analysis of 46 studies from 19 countries on the host-specific genetic factors in humans that were associated with infection of SARS-CoV-2 (cases defined as individuals with laboratory-confirmed infection of SARS-CoV-2, clinical diagnosis of COVID-19, or those who had relevant electronic health records/International Classification of Diseases (ICD) coding of COVID-19 diagnosis, or those with self-reported COVID-19 irrespective of their symptoms), COVID-19 hospitalization (cases referred to individuals who were hospitalized due to symptoms of laboratory-confirmed infection of SARS-CoV-2) and COVID-19 severity (critically ill cases were defined as individuals who (1) were hospitalized due to symptoms of laboratory-confirmed infection of SARS-CoV-2 and (2) required respiratory support; or (3) died due to COVID-19-associated causes) [18]. With the general population as controls, SARS-CoV-2 infection, COVID-19 hospitalization and severity were adopted as the outcomes of interest in the current MR study. Notably, biases could result if both the case and control participants in the outcome data set are also in the exposure data set [19]. Thus, we selected data sources by minimizing the chance of sample overlap in the exposure and outcome data sets. For instance, both the GWAS meta-analysis of education attainment [16] and intelligence [17] comprised participants from the U.K. Biobank. Although the COVID-19 HGI has released larger GWAS meta-analysis data sets of infection, hospitalization and severity consisting of samples from the U.K. Biobank, we adopted the data sets that excluded the U.K. Biobank participants to avoid any potential biases due to sample overlap.

If a significant causal association was observed in univariable MR analysis, the potential mediating mechanism was evaluated using multivariable MR analysis [20,21]. The potential mediators investigated included body mass index (BMI), smoking, reduced leisure-time physical activity and coronary artery disease (CAD) (elaborated in Supplementary Table S1). As education attainment and intelligence were reported to influence the health outcomes independently and jointly [14], intelligence was also tested as a potential mediator in the pathway from education attainment to COVID-19 outcomes. We hypothesized that the underlying mechanisms from intelligence to COVID-19 outcomes may be similar to that for education attainment, and we tested this using multivariable MR analysis. The same applies to education attainment in the association between intelligence and COVID-19. Data sources of exposures, potential mediators and outcomes are listed in Table 1.

### 2.2. MR analyses

The selection of genetic instruments and data harmonization are detailed in Supplementary Methods S1 and S2, respectively. Univariable inverse-variance weighted (IVW) method was used for main MR analysis to assess the total effect of the exposure on the outcome [20,22]. Weighted median method, MR-Egger regression and contamination mixture method were employed as sensitivity analyses. MR-Egger intercept test and global test of MR-PRESSO were applied to detect the presence of pleiotropy. Multivariable IVW analysis was also performed to dissect the mechanisms in the causal pathway from the risk factor to the outcome [20,21]. While the causal estimates derived from univariable MR analysis represent the total effect of the exposure on the outcome, multivariable MR analysis can be used to estimate the direct causal effect of the exposure on the outcome by keeping the potential mediators constant. The presence of a difference between the causal estimates of the univariable (total effect) and multivariable MR analysis (direct causal effect) implies that causal effect acts at least in part via the potential mediator (indirect effect) [20]. A multivariable MR-Egger intercept test was applied to detect the presence of residual pleiotropy via other unmeasured risk factors. Different methods of MR analyses and power calculation are described in Supplementary Methods S3 and S4, respectively.

**Table 1:** Data sources used in the Mendelian randomization analyses.

	Trait	Exposure/Outcome/Potential Mediator in MR Analyses?	Description of Data Source	Ancestry	Sample Size
1	Education attainment [16]	Exposure/potential mediator	A meta-analysis of 71 independent GWAS of education attainment, which was defined as the number of years of schooling that the study participants completed. Proxies were identified from the publicly available summary statistics, which excluded samples from 23andme due to data restriction.	European	1,131,881
2	Intelligence [17]	Exposure/potential mediator	A meta-analysis of GWAS of intelligence from 14 cohorts. Although each cohort adopted different measures of intelligence, all cohorts were operationalized to index a common latent g factor that underlies different dimensions of cognitive functioning.	European	269,867
3	SARS-CoV-2 infection [18]	Outcome	The SARS-CoV-2 infection cases were defined as individuals with laboratory-confirmed infection of SARS-CoV-2, clinical diagnosis of COVID-19, or those who had relevant electronic health records/ICD coding of COVID-19 diagnosis, or those with self-reported COVID-19 irrespective of their symptoms. Due to the potential overlap of samples from the exposure and outcome data set, we used the summary statistics of COVID-19 susceptibility, in which the U.K. Biobank participants were excluded. In addition, due to data restrictions, the summary statistics applied in this study also excluded samples from the 23andme cohort. European-only summary statistics were used in MR analysis.	European	32,494 cases; 1,316,207 controls
4	COVID-19 hospitalization [18]	Outcome	The hospitalized COVID-19 patients were defined as individuals who were hospitalized due to symptoms of laboratory-confirmed infection of SARS-CoV-2. The controls were from the general population. Due to the potential overlap of samples from the exposure and outcome data set, we used the summary statistics of COVID-19 hospitalization in which the U.K. Biobank participants were excluded. In addition, due to data restrictions, the summary statistics applied in this study also excluded samples from the 23andme cohort. European-only summary statistics were used in MR analysis.	European	8316 cases; 1,549,095 controls
5	COVID-19 severity [18]	Outcome	The critically ill COVID-19 cases were defined as individuals who (1) were hospitalized due to symptoms of laboratory-confirmed infection of SARS-CoV-2 and (2) required respiratory support; or (3) died due to COVID-19-associated causes. The controls were from the general population. Due to the potential overlap of samples from the exposure and outcome data set, we used the summary statistics of COVID-19 severity in which the U.K. Biobank participants were excluded. In addition, due to data restrictions, the summary statistics applied in this study also excluded samples from the 23andme cohort. European-only summary statistics were used in MR analysis.	European	4792 cases; 1,054,664 controls
6	Coronary artery disease [23]	Potential mediator	A meta-analysis of 48 GWAS of coronary artery diseases of CARDIoGRAMplusC4D Consortium.	Predominantly European (77%)	60,801 cases; 123,504 controls
7	Body mass index (BMI) [24]	Potential mediator	A meta-analysis of U.K. Biobank data with a previous GWAS of the Genetic Investigation of ANthropometric Traits (GIANT) consortium.	European	694,649
8	Overall activity time (measurement based on wrist-worn accelerometer) [25]	Potential mediator	A GWAS of overall activity time (a continuous phenotype) conducted in the U.K. Biobank participants with wrist-worn accelerometer.	European	91,105
9	Smoking status (ever regular vs. never regular) [26]	Potential mediator	A meta-analysis of 35 GWAS of multiple stages of tobacco use and alcohol use. Only the summary statistics related to tobacco use were adopted in the current MR study, as we aim to test if tobacco use is a potential mediator in the causal pathway from education attainment/intelligence.	European	1,232,091

## 3. Results

### 3.1. Two-Sample MR of Education Attainment on SARS-CoV-2 Infection, COVID-19 Hospitalization and Severity

**Figure 2:**
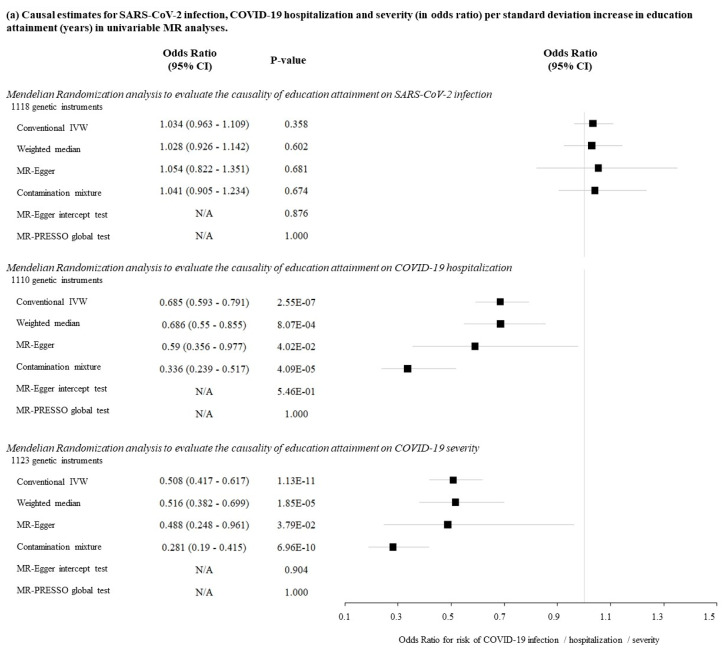

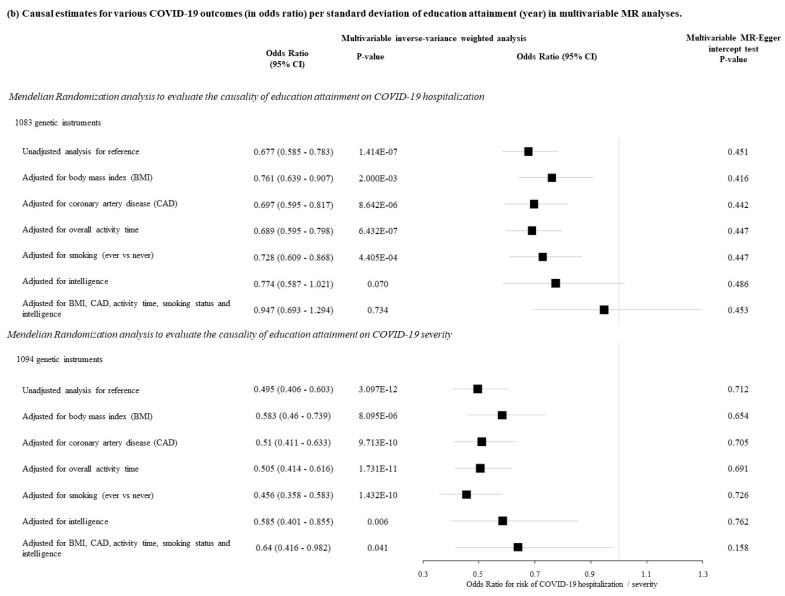
Results of Mendelian randomization analysis in evaluating the causal association between education attainment and SARS-CoV-2 infection, COVID-19 hospitalization and severity. (**a**) Causal estimates for SARS-CoV-2 infection, COVID-19 hospitalization and severity (in odds ratio) per standard deviation increase in education attainment (years) in univariable MR analyses. (**b**) Causal estimates for COVID-19 hospitalization and severity (in odds ratio) per standard deviation of education attainment (year) in multivariable MR analyses.

Univariable MR analysis demonstrated that genetically determined education attainment had null causal association with SARS-CoV-2 infection (IVW: odds ratio (OR) = 1.034; 95% confidence interval (CI): 0.963–1.109; *p* = 0.358; Figure 2a). Conversely, education attainment had an inverse association with COVID-19 hospitalization (IVW: OR = 0.685 per 1 SD increase in years of schooling (~4.2 years); 95% CI: 0.593–0.791; *p* < 0.001) and COVID-19 severity (IVW: OR = 0.508; 95% CI: 0.417–0.617; *p* < 0.001). Similar estimates were obtained from the weighted median method, MR-Egger regression and contamination mixture method (Figure 2a). The MR-Egger intercept and MR-PRESSO global tests were insignificant (Figure 2a).

With the multivariable MR approach, there was little change in causal estimate for COVID-19 hospitalization after individual adjustment for the beta estimates of BMI, CAD, overall activity time, and smoking status. However, the causal association of education attainment with COVID-19 hospitalization was attenuated after adjustment for the beta estimates of intelligence (OR = 0.774; 95% CI: 0.587–1.021; *p* = 0.07; Figure 2b). Upon adjustment for all the five potential mediators at the same time, the association was also attenuated (OR = 0.947; 95% CI: 0.693–1.294; *p* = 0.73; Figure 2b). For COVID-19 severity, little change in causal estimate was observed after adjusting for each of the five potential mediators, as well as adjusting for all the potential mediators at the same time (Figure 2b). All the multivariable MR-Egger intercept tests were insignificant (Figure 2b).

### 3.2. Two-Sample MR of Intelligence on SARS-CoV-2 Infection, COVID-19 Hospitalization and Severity

**Figure 3:**
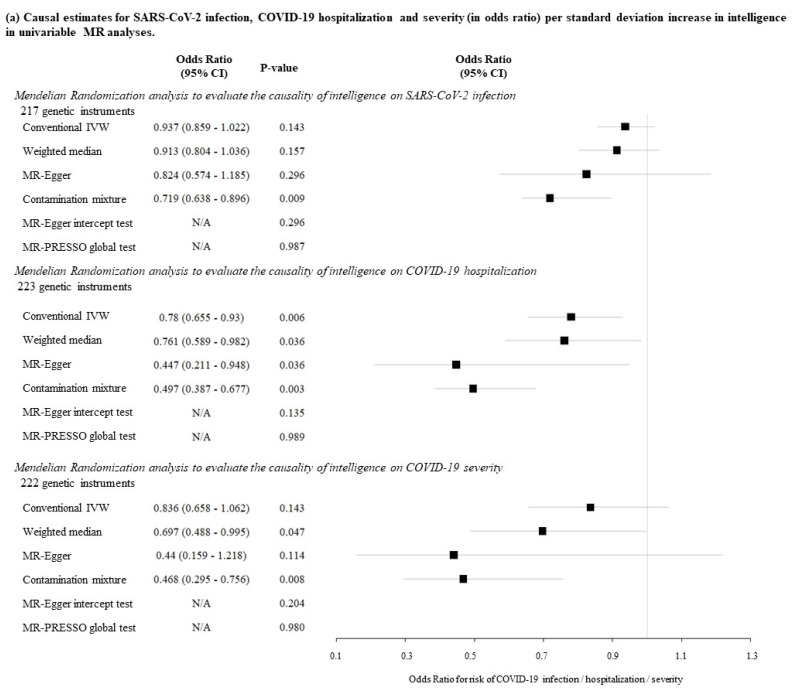

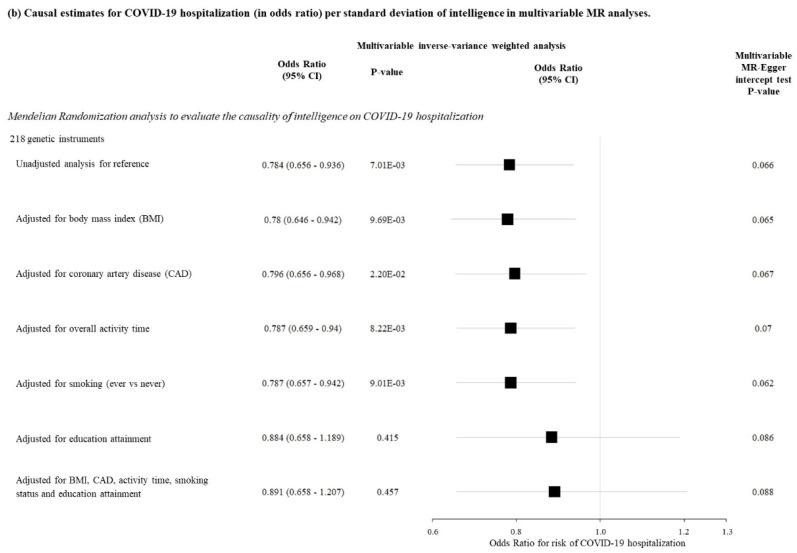
Results of Mendelian randomization analysis in evaluating the causal association between intelligence and SARS-CoV-2 infection, COVID-19 hospitalization and severity. (**a**) Causal estimates for SARS-CoV-2 infection, COVID-19 hospitalization and severity (in ods ratio) per standard deviation increase in intelligence in univariable MR analyses. (**b**) Causal estimates for COVID-19 hospitalization (in odds ratio) per standard deviation of intelligence in multivariable MR analyses.

Univariable IVW analysis showed null causal association of genetically determined intelligence with SARS-CoV-2 infection (OR = 0.937; 95% CI: 0.859–1.022; *p* = 0.143), with similar null association observed in sensitivity analyses (Figure 3a). While univariable IVW analysis suggested that genetically higher intelligence was causally associated with reduced risk of COVID-19 hospitalization (for each SD increase in general intelligence, OR = 0.780; 95% CI: 0.655–0.930; *p* = 0.006), similar significant association was observed in the sensitivity analyses (Figure 3a). For COVID-19 severity, potential inverse causal association was observed in weighted median method (OR = 0.697; 95% CI: 0.488–0.995; *p* = 0.047) and contamination mixture method (OR = 0.468; 95% CI: 0.295–0.756; *p* = 0.008; Figure 3a), but not for the main IVW analysis. All the univariable MR-Egger intercept and MR-PRESSO global tests for the above analyses were insignificant.

In multivariable MR analysis, little change in causal estimates for COVID-19 hospitalization was observed after individually adjusting for the beta estimates of BMI, CAD, overall activity time and smoking status (Figure 3b). The causal association was attenuated after adjustment for education attainment (OR = 0.884; 95% CI: 0.658–1.189; *p* = 0.415; Figure 3b). Similarly, upon adjustment for all the five potential mediators at the same time, the association was attenuated (OR = 0.891; 95% CI: 0.658–1.207; *p* = 0.457; Figure 3b). The multivariable MR-Egger intercept tests were all insignificant (Figure 3b).

## 4. Discussion

To the best of our knowledge, this is the first MR study to date that examines the causal relationship of genetic predisposition to higher education attainment and intelligence with SARS-CoV-2 infection, COVID-19 hospitalization and severity, with an attempt to dissect the underlying mechanisms. We revealed a causal relationship of genetic predisposition to higher education attainment and intelligence with reduced risk of COVID-19 hospitalization and/or severity. While the causal pathway from education attainment to COVID-19 hospitalization may be mediated by intelligence, education attainment may have an independent role on COVID-19 severity. Meanwhile, education attainment is a mediator in the causal pathway between genetically increased intelligence and lower risk of COVID-19 hospitalization. Null causal association was observed for genetically determined education attainment and intelligence with SARS-CoV-2 infection.

Our analysis unraveled the causal effects of genetically increased education attainment on reduced COVID-19 hospitalization and severity, with robust evidence in both the main and sensitivity analyses. In the GWAS meta-analysis of COVID-19 severity, individuals were considered as cases if their deaths were due to COVID-19-associated causes. Thus, our study finding implied that genetic predisposition to higher education attainment may causally lower the risk of COVID-19-associated deaths, which is partially in line with most of the published observational studies in the United States [8], Sweden [10] and Peru [9]. In the National Health and Nutrition Examination Survey (NHANES), individuals with education lower than high school level (11.2% of the 2017–2018 NHANES sample) were overrepresented and accounted for approximately 25% of the COVID-19 deaths [11]. Our analysis also revealed an inverse causal effect of genetically determined education attainment on COVID-19 hospitalization, which is consistent with the finding from the only available observational study conducted using electronic medical records serving Eastern Massachusetts of the United States [7]. While all the above observational studies treated education attainment as a categorical variable and classified it into several levels, education attainment in our study was a continuous variable representing the number of years of schooling. Thus, the magnitude of association derived from previous observational studies and this MR study cannot be directly compared. It also came to our attention that Yoshikawa et al. published a univariable MR analysis very recently by making use of a smaller number of 235 genetic instruments for education attainment [27] extracted from the latest meta-analysis but a reduced data set comprising 766,345 individuals after excluding the 23andMe samples. In contrast, our current study had higher statistical power due to the use of more than 1110 independent SNPs derived from the same but complete data set consisting of 1,131,881 individuals as genetic instruments [16]. In addition, they did not conduct multivariable MR analyses to uncover the potential mechanisms, and they did not examine the causal effects of education attainment on SARS-CoV-2 infection and COVID-19 hospitalization [27]. Likewise, the causal association of intelligence with COVID-19 outcomes was not examined in the study conducted by Yoshikawa et al.

In this study, we also demonstrated a causal association of genetically higher intelligence with reduced COVID-19 hospitalization, while the association with reduced COVID-19 severity was inconsistent. To our knowledge, no observational studies have investigated the association of intelligence with COVID-19 outcomes. Notably, the attenuation of association after adjustment for education attainment suggested that the causal effects of genetically higher intelligence on a lower risk of COVID-19 hospitalization were mediated via education attainment. This implied that individuals with genetically higher intelligence, i.e., those with higher cognitive reasoning abilities, may not necessarily have a lower risk of COVID-19 hospitalization if they did not receive adequate education. Conversely, the causal association of genetically higher education attainment with lower risk of COVID-19 severity remained significant even after adjustment for intelligence, as well as adjustment for all the five potential mediators at the same time, suggesting that higher education attainment had independent protective effects on the progression of COVID-19 to severe forms, which was even independent of the joint effect of all the five potential mediators. Taken together, higher education attainment might reduce the risk of COVID-19 outcomes both independently and jointly with intelligence. Indeed, empirical evidence in the public health literature has characterized levels of formal education as a primary socioeconomic factor that determines the health outcomes of people amid large-scale public health emergencies. In times of crisis such as the Middle East respiratory syndrome [28] and COVID-19 [29,30], individuals with higher education attainment demonstrated better preparedness and adaptiveness in peculiar and unfamiliar situations by showcasing a higher level of knowledge and health literacy, adopting self-care and risk-avoidance behavior, and having increased awareness of the need to seek medical advice timely. Furthermore, education attainment is associated with individuals’ information-seeking and acquisition capability. Along with the COVID-19 pandemic, related misinformation and conspiracy theories were widespread through informal personal networks or via social media platforms [31]. Such phenomena were known as “infodemic”, aggravating confusion and distrust in public and creating resistance to mitigation efforts. In particular, individuals with lower education levels might not possess adequate ability to discern the accuracy of information sources [32] and may be more prone to be overloaded by the vast quantity of inconsistent information about COVID-19, resulting in information anxiety and avoidance that further prohibits them from obtaining authentic and timely information on health advice and protective measure [33]. All these offer plausible explanations to the findings in our study, supporting that higher education attainment may reduce the risk of hospitalization or severe complications due to COVID-19.

This MR study could not provide sufficient evidence to support the presence of a causal relationship of genetically determined education attainment and intelligence with SARS-CoV-2 infection. While observational studies demonstrated that individuals with lower education attainment had an increased risk of testing positive for SARS-CoV-2 infection [12,13], one possible explanation is that observational studies are subjected to residual confounding. Although these two observational studies have adjusted for other social determinants, they may have omitted some important confounders. An example is the number of people per unit of living area, as cramped living condition disables social distancing and may increase the risk of SARS-CoV-2 infection [34]. Notably, SARS-CoV-2 infection is largely dependent on contact with an infected person or transmission via respiratory droplets, which may be unavoidable, especially due to the high prevalence of asymptomatic cases of COVID-19 (approximately 40%–45%) [35] and inevitable contact within the same family. While MR strategy uses genetic instruments to represent lifelong exposure to a risk factor, it does not take into account the short-term exposure to the virus. Further investigations with different study designs are required to assess the causal relationship of education attainment and intelligence with SARS-CoV2-infection.

This study has important implications. Evidence of socioeconomic inequality was observed in relation to COVID-19-related outcomes, with a higher risk of SARS-CoV-2 infection and COVID-19 mortality among the deprived group [2]. Our findings suggested that the COVID-19 outcomes may be improved by prioritizing education as a non-pharmacological intervention in combating COVID-19. In particular, “infodemic” has led to mass suspicion over information about COVID-19. People who believe in conspiracy theories and misinformation had increased vaccine hesitancy [6,36,37,38,39,40], increased support for the controversial treatment against COVID-19 (hydroxychloroquine) [39,40], as well as poorer engagement in health-protective behavior against COVID-19 [37,38,41]. Meanwhile, they were also reported to have lower education levels [4,5,42]. Notably, vaccination did not only protect individuals against the infection of SARS-CoV-2, but vaccines such as Pfizer-BioNTech BNT162b2 or ChAdOx1-S could also reduce the risk of COVID-19 related hospitalization [43,44], severe or critical COVID-19 related hospitalization [44], and mortality [43,44]. In a randomized clinical trial (RCT), there was no difference in the clinical status of hospitalized patients at day 14 who received hydroxychloroquine compared to those who received a placebo [45]. A meta-analysis of RCT even demonstrated that treatment with hydroxychloroquine was linked to an increased risk of death among COVID-19 patients [46]. Since individuals susceptible to conspiracy theories and misinformation were less likely to be vaccinated and intended to support the use of hydroxychloroquine, they might have more severe outcomes if they were infected with SARS-CoV-2. Moreover, higher education attainment not only enhances the knowledge of the population but also benefits the choice of a healthy lifestyle, subsequently improving health outcomes of the population amid the COVID-19 pandemic and likely alleviating the burden brought to the stretched healthcare system. In addition to addressing the causation of education attainment on COVID-19-related outcomes, our study offers insights into identifying the population susceptible to the adverse outcomes. While prolonging the length of education among the general population is a complicated and resource-intensive process, it is imperative to devise timely strategies to address the needs of people with lower education levels during the pandemic. Although national governments and health authorities have developed guidelines or recommendations for infection prevention practices, adherence to these guidelines among individuals with lower education levels could be uncertain if they were not equipped with adequate knowledge and correct attitude toward the suggested practices [32,33]. As individuals with lower education levels are more inclined to obtain COVID-related information from informal channels, such as social media platforms, rather than official sources or news organizations [47], tailoring of teaching materials and guidelines that fit the needs and expectations of less-educated individuals, such as preparing more laymen materials to be disseminated through the social media platforms of popular opinion leaders, may be required. The policymakers may also consider reserving appropriate and sufficient resources to meet the healthcare and social needs of individuals in the lower socioeconomic group, especially those with lower education attainment, during the pandemic. This is particularly essential as limited access to healthcare services under the containment policies of COVID-19, together with the material and social deprivation of these underprivileged individuals, may further worsen their health outcomes.

A major strength of this study is the use of two-sample MR analysis with minimal sample overlap, which was reported to provide a less biased causal estimate than one-sample MR analysis [19]. Yet, there was sample overlap between the exposure and outcome GWAS (<5.87%; Table 2). With the increase in sample overlap, bias toward the confounded association might be present [19]. In view of this, we estimated the bias and type I error rate under the null model that arose due to sample overlap. Assuming the bias of the observational estimate was 0.4 log odds ratio of the outcome per SD increase in the exposure, the maximum bias caused by sample overlap was estimated to be 0.001, which was likely negligible, while the type I error rate was 0.05 (Table 2). This could be attributed to the strong instruments, as revealed by the relatively high F-statistics (Table 2). The causal relationship identified is likely to be genuine. Moreover, the inverse causal effect of genetically higher education attainment and intelligence on decreased risk of COVID-19 hospitalization and/or severity were supported by multiple sensitivity analyses on the basis of different assumptions, thus providing robust evidence on the causality. Furthermore, due to the large sample size of the GWAS meta-analysis from which the summary statistics were retrieved from, our MR analysis is well-powered (Supplementary Figure S1).

There are also limitations. First, the genetic instruments may act on the outcome via some unknown pathways other than the exposure, violating the MR assumptions. We thus adopted MR-Egger intercept and MR-PRESSO global tests to detect horizontal pleiotropy. Both the tests were insignificant in all the analyses, suggesting that horizontal pleiotropy is unlikely, although this cannot be ruled out unequivocally. Second, the GWAS meta-analysis conducted by COVID-19 HGI was subjected to selection bias [18]. As individuals with higher socioeconomic status (as indicated by higher education attainment) might have better access to the healthcare system, they might be more easily diagnosed with COVID-19, especially during the early phase of the pandemic. They might be overrepresented as cases in the GWAS meta-analysis of SARS-CoV-2 infection. Such selection bias might have distorted the original intention to examine the relationship between genetic variation and SARS-CoV-2 infection since genetic association with education attainment inevitably played a role. Third, the current MR analysis was conducted using the summary statistics of GWAS meta-analysis conducted among the Europeans, indicating that the causality inferred may only be applicable to Europeans only. Whether our findings and policy implications could be generalized to other ethnicities will require further investigation. Fourth, in multivariable MR analysis, we were unable to provide the conditional F-statistics as illustrated by a very recent publication implemented by the MVMR package in R [48]. As we lacked individual-level data, and we did not estimate the gene-exposure association in separate non-overlapping samples, we could not calculate the pairwise covariance between each genetic instrument with any two exposures for all genetic instruments and all exposures. Further information is required for accurate estimates of conditional F-statistics. Fifth, we attempted to identify the potential mediators in the causal pathway by multivariable MR analysis. Nevertheless, the list of potential mediators included is not exhaustive. Further investigation with additional potential mediators is required to dissect the causal pathway.

In conclusion, genetic predisposition to higher education attainment was causally and independently associated with reduced risk of COVID-19 severity. This study provides insights on the potential alleviation of COVID-19-related health inequality by reduction in socioeconomic inequality via education.

## Figures and Tables

**Table 2:** Strength of genetic instruments in each of the Mendelian randomization analyses.

	MR Analysis	Exposure		Outcome		Maximum Percentage of Sample Overlap #	No. of Genetic Instruments in MR Analysis (No. of Independent Genome-Wide SNPs Identified from GWAS - No. of Instruments without Proxies - No. of Proxies that Could Not Reach Genome-Wide Significance - No. of Pleotropic Outliers Identified by Radial MR)	Variance Explained by the Instruments on Exposure (%)	F-Statistics (Average per Instrument)	Bias due to Sample Overlap	Type I Error due to Sample Overlap
		Trait	No. of Samples	Trait	No. of Samples						
1	Univariable MR analysis	Education attainment	1,131,881	SARS-CoV-2 infection	32,494 cases; 1,316,207 controls	5.87%	1118 (1271 - 24 - 56 - 73)	5.22	55.7	0.001	0.05
2				COVID-19 hospitalization	8316 cases; 1,549,095 controls	3.94%	1110 (1271 - 27 - 59 -75)	5.20	55.88	0	0.05
3				COVID-19 severity	4792 cases; 1,054,664 controls	1.37%	1123 (1271 - 21 - 57 - 70)	5.25	55.79	0	0.05
4	Univariable MR analysis	Intelligence	269,867	SARS-CoV-2 infection	32,494 cases; 1,316,207 controls	0.02%	217 (242 - 1 - 2 - 22)	3.40	43.74	0	0.05
5				COVID-19 hospitalization	8316 cases; 1,549,095 controls	0%	223 (242 - 3 - 2 - 14)	3.46	43.34	NA	NA
6				COVID-19 severity	4792 cases; 1,054,664 controls	0%	222 (242 - 1 - 2 - 17)	3.44	43.27	NA	NA
7	Multivariable MR analysis	Education attainment	1,131,881	COVID-19 hospitalization	8316 cases; 1,549,095 controls	3.94%	1083 (1271 - 49 - 66 - 73)	5.07	55.76 *	0	0.05
8				COVID-19 severity	4792 cases; 1,054,664 controls	1.37%	1094 (1271 - 47 - 65 - 65)	5.12	55.78 *	0	0.05
9	Multivariable MR analysis	Intelligence	269,867	COVID-19 hospitalization	8316 cases; 1,549,095 controls	0%	218 (242 - 5 - 3 - 16)	3.39	43.4 *	NA	NA

# The percentage of sample overlap between the exposure and outcome data sets is taken with respect to the larger data set [19]. * Refer to the unconditional F-statistics.

## Data Availability

Summary statistics of genetic instruments can be obtained from the websites listed on the referenced GWAS or GWAS meta-analysis.

## Supplementary Materials

The following are available online at https://www.mdpi.com/article/10.3390/jcm10214870/s1, Supplementary Methods S1: Selection of genetic instruments for Mendelian randomization (MR) analysis, Supplementary Methods S2: Data harmonization, Supplementary Methods S3: Mendelian randomization analyses, Supplementary Methods S4: Calculation of power, F-statistics, bias and Type I error rate due to sample overlap, Table S1: Potential mediators between education attainment and COVID-19 outcomes, Figure S1: Power calculation of MR analyses.
