# Supplementary material for: Education Attainment, Intelligence and COVID-19: A Mendelian Randomization Study
Source: J Clin Med. 2021 Oct 22;10(21):4870. doi: 10.3390/jcm10214870 (PMC8584527; doi:10.3390/jcm10214870)
Supplement: Supplementary file 1 [file jcm-10-04870-s001.zip › jcm-1406123-supplementary.pdf]

Supplementary Material

Supplementary Methods 1. Selection of genetic instruments for Mendelian randomization (MR) analysis.

Supplementary Methods 2. Data harmonization.

Supplementary Methods 3. Mendelian randomization analyses.

Supplementary Methods 4. Calculation of power, F-statistics, bias and Type I error rate due to sample overlap.

Supplementary Table 1. Potential mediators between education attainment and COVID-19 outcomes.

Supplementary Figure 1. Power calculation of MR analyses.

(a) Power of univariable MR analysis in evaluating the causal association between genetically determined education attainment and SARS-CoV-2 infection.
(b) Power of univariable MR analysis in evaluating the causal association between genetically determined education attainment and COVID-19 hospitalization.
(c) Power of univariable MR analysis in evaluating the causal association between genetically determined education attainment and COVID-19 severity.
(d) Power of univariable MR analysis in evaluating the causal association between genetically determined intelligence and SARS-CoV-2 infection.
(e) Power of univariable MR analysis in evaluating the causal association between genetically determined intelligence and COVID-19 hospitalization.
(f) Power of univariable MR analysis in evaluating the causal association between genetically determined intelligence and COVID-19 severity.
(g) Power of multivariable MR analysis in evaluating the causal association between genetically determined education attainment and COVID-19 hospitalization.
(h) Power of multivariable MR analysis in evaluating the causal association between genetically determined education attainment and COVID-19 severity.
(i) Power of multivariable MR analysis in evaluating the causal association between genetically determined intelligence and COVID-19 hospitalization.

Supplementary Methods 1. Selection of genetic instruments for Mendelian randomization (MR) analyses.

Genome-wide independent genetic variants associated with the exposure, which were derived from the largest public available GWAS / GWAS meta-analysis and not in linkage disequilibrium (LD; r^2<0.1) with each other, were initially selected as the genetic instruments to represent the genetically determined exposure trait. For example, the 1,271 and 242 genome-wide independent variants [1, 2] were selected as the initial genetic instruments for number of years of schooling and intelligence, respectively. The summary statistics of these initial genetic instruments were retrieved from the datasets of outcome (for both univariable and multivariable MR) and potential mediators (for multivariable MR only). If an initial genetic instrument was unavailable in the GWAS of outcome or potential mediator datasets, a proxy variant in high LD ($r^2 \geq 0.8$) and present in all the exposure, outcome and potential mediator (for multivariable MR only) datasets was selected as the genetic instrument to replace the initial one. If the proxy was no longer associated with the exposure with genome-wide significance (threshold of P: $5x10^{-8}$) or no proxies could be identified, the genetic instrument was excluded from MR analysis. Radial MR analysis was applied to identify outliers which had large contribution to Cochran's Q statistics that might imply heterogeneity, including horizontal pleiotropy [3]. Upon exclusion of these outlying genetic instruments, MR analyses were subsequently performed.

Supplementary Methods 2. Data harmonization.

All the genetic instruments were oriented such that the effect alleles were positively associated with the exposure. The effect alleles were matched across the summary data of the exposure, potential mediator and outcome dataset. For palindromic genetic instruments, effect allele frequencies were used to align them on ambiguous strands across different datasets. If the minor allele frequencies of the palindromic instruments were greater than 0.3, a proxy in high LD will be identified for the instrument to avoid ambiguity [4].

Supplementary Methods 3. Mendelian randomization (MR) analyses.

Radial MR analysis was a modification of the traditional scatter plot to facilitate the visualization and detection of outliers, which adopted Cochran's Q statistic to assess heterogeneity, as excessive heterogeneity might imply violation of the MR assumptions, including horizontal pleiotropy [3]. Using the modified second-order weight, genetic instruments with large contributions to Cochran's Q statistic were considered outliers [3] and they were excluded from subsequent MR analyses.

Univariable inverse-variance weighted (IVW) [5] method was used as the main MR analysis to assess the total effect of the exposure on the outcome [6]. While IVW is the conventional method applied in MR analysis, this method is limited by the assumption that all instrumental variables are valid. To address this issue, weighted median method was used as a sensitivity analysis as provides consistent estimates even when up to 50% of the information comes from invalid instrumental variables [7]. On the other hand, based on the assumption that the magnitude of the pleiotropic effects are independent of the SNP-risk factor associations across all variants [Instrument Strength Independent of Direct Effect (InSIDE) assumption], the slope coefficient from MR-Egger regression gave a consistent estimate of causal effect even if all the genetic variants are invalid instrumental variables, thus serving as another sensitivity analysis [8]. Meanwhile, the contamination mixture method was based on the "plurality valid" assumption that among various groups of genetic instruments having the same asymptotic causal estimate, the largest group should be the group of valid instruments [9]. This method firstly identified groups of genetic instruments with similar estimates, suggesting potential distinct mechanisms from the exposure to the outcome for different groups

[9]. The method subsequently performed robust MR analysis which had well-controlled Type I error rates with up to 50% invalid instruments [10].

To test for the presence of pleiotropy, MR-Egger intercept [8] and MR pleiotropy residual sum and outlier (MR-PRESSO) [11] tests were adopted. Based on the InSIDE assumption [8], the intercept of MR-Egger regression represents the average pleiotropic effects across all SNPs. Meanwhile, there are three components in MR-PRESSO analysis: (i) global test has sufficient power to evaluate the overall horizontal pleiotropy among all instruments even if pleiotropy just occurs in less than half of the instruments; (ii) outlier test provides the causal estimate upon removal of pleiotropic genetic instruments; and (iii) distortion test determines if there is any significant difference in the causal estimate before and after the removal of pleiotropic genetic instruments [11]. In the current analysis, the MR-PRESSO global test was applied to examine the presence of overall horizontal pleiotropy, after outliers identified by Radial MR analysis were excluded from the analysis.

In case univariable MR analysis revealed a causal association between the exposure and outcome, multivariable IVW analysis was also performed to dissect the mechanisms in the causal pathway from the exposure to the outcome [6, 12]. The direct causal effect of the exposure on the outcome was evaluated by multivariable IVW by adjusting for the beta estimates of potential mediators. If the multivariable IVW analysis yielded an estimate which is deviated from the null, this implies the exposure is independently associated with the outcome that keeping the potential mediators unchanged would not affect the outcome [12]. On the contrary, attenuation of causal

estimates to null upon adjustment for beta estimates of potential mediators implies that the mediators play a role in the causal pathway that keeping them constant would affect the outcome. Multivariable MR-Egger intercept test was utilized to detect for presence of residual pleiotropy via other unmeasured risk factors [13].

While weighted median method, contamination mixture method, univariable and multivariable IVW and MR-Egger analyses were conducted with the 'MendelianRandomization' package in R [14], radial MR and MR-PRESSO analysis were performed using "RadialMR" [3] and "MRPRESSO" [11] packages respectively.

Supplementary Methods 4. Calculation of power, F-statistics, bias and Type I error rate due to sample overlap

The proportion of variance explained by each genetic instrument on the exposure trait was calculated from the formula: 2x(minor allele frequency)x(1-minor allele frequency)x(effect size)^2, where effect size is in standard deviation (SD) of the exposure. The bias and Type I error rate due to sample overlap under the null model were estimated using a web application (https://sb452.shinyapps.io/overlap) [15]. The unconditional F-statistics for univariable MR analysis, a measure of strength of genetic instruments, and the number of genetic instruments applied in each MR analysis, were also computed using the same web tool [15]. The figures are included in Table 2. An online tool (https://sb452.shinyapps.io/power/) [16] was employed to calculate the statistical power (Supplementary Figure 1).

Supplementary Table 1. Potential mediators between education attainment and COVID-19 outcomes.

Potential mediators	Reference
**Body Mass Index (BMI)**	
In Mendelian randomization analysis, 1 standard deviation longer education (3.6 years) was associated with reduced BMI by 0.17.	[17]
High BMI ($\geq$30 kg/m^2) or obesity affects both the respiratory and immune system by multiple mechanisms, such as secreting cytokine and adipokine from adipose tissue to trigger the pro-inflammatory status in obese individuals, increasing their risk of thrombosis, incoordination of innate and adaptive immune responses, insufficient response to antibody, and cytokine storm.	[18]
**Smoking (ever vs never)**	
In Mendelian randomization analysis, 1 standard deviation longer education (3.6 years) was associated with 35% reduced odds of smoking (OR=0.65).	[17]
Smoking was reported to inhibit degradation of angiotensin II, resulting in overactivity of local angiotensin II, which played a role in inflammatory and thrombotic complications of COVID-19.	[19]
**Leisure time physical activity**	
In a national cross-sectional survey in UK, lower education attainment was associated with lower weekly leisure time physical activity.	[20]
Physical activity involved skeletal muscle contraction and released muscle-derived cytokines, triggering anti-inflammatory response and had positive effects on the immune system, thus reducing the severity of viral infections, including COVID-19.	[21]
**Coronary artery disease (CAD)**	
In prospective analysis, increase in 1 standard deviation of education (3.6 years) was associated with 20% reduced odds of incident coronary heart disease (OR=0.80). There was a dose response relationship between years of education and risk of coronary heart disease.<br>In conventional Mendelian randomization analysis, 1 standard deviation increase in education years was associated with 33% reduced odds of coronary heart disease (OR=0.67).	[17]
Individuals with CAD were found to have hyperinflammatory response towards the infection of SARS-CoV-2.	[22]

Supplementary Figure 1. Power calculation of MR analyses.

(a) Power of univariable MR analysis in evaluating the causal association between genetically determined education attainment and SARS-CoV-2 infection.

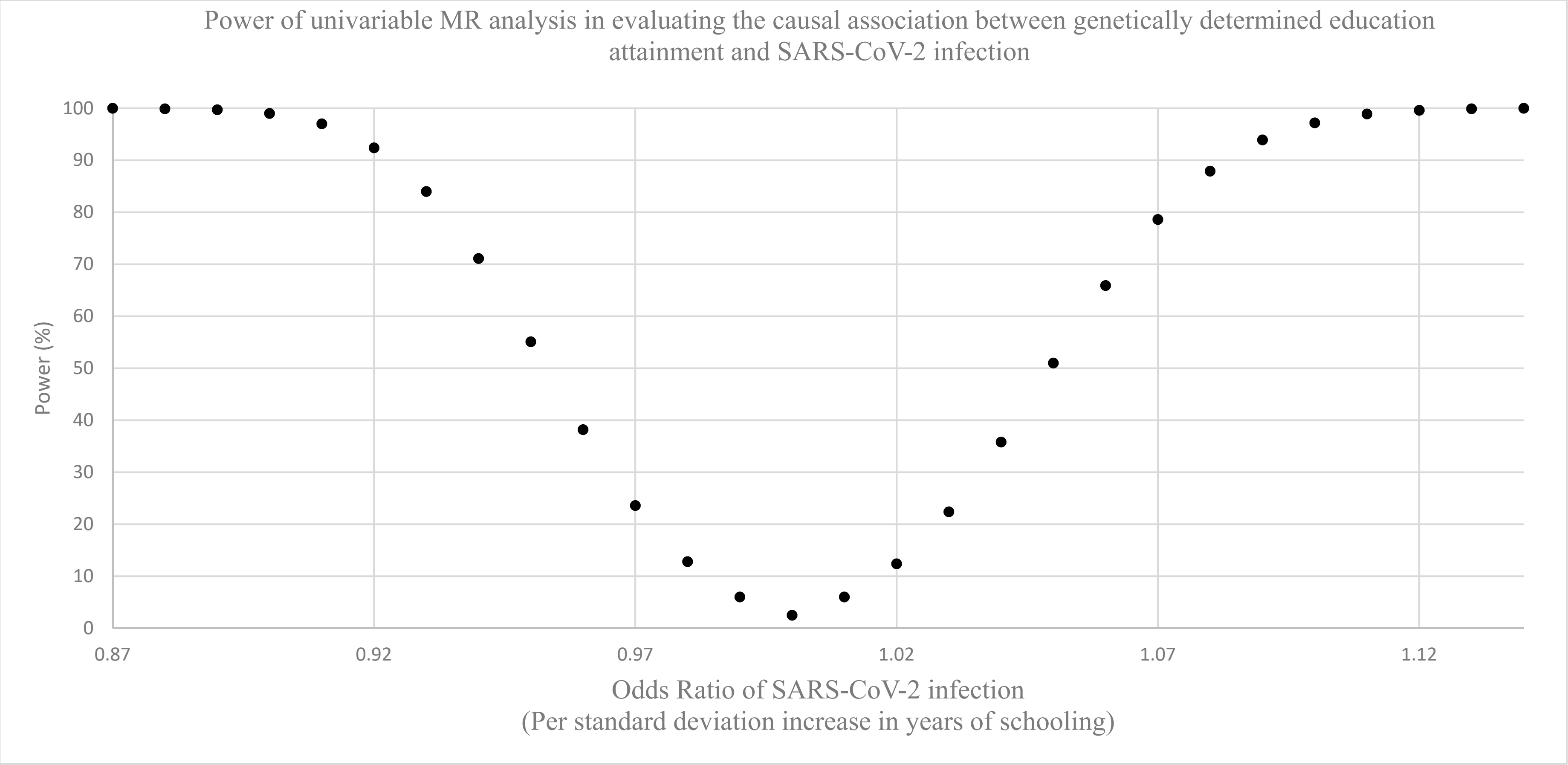

(b) Power of univariable MR analysis in evaluating the causal association between genetically determined education attainment and COVID-19 hospitalization.

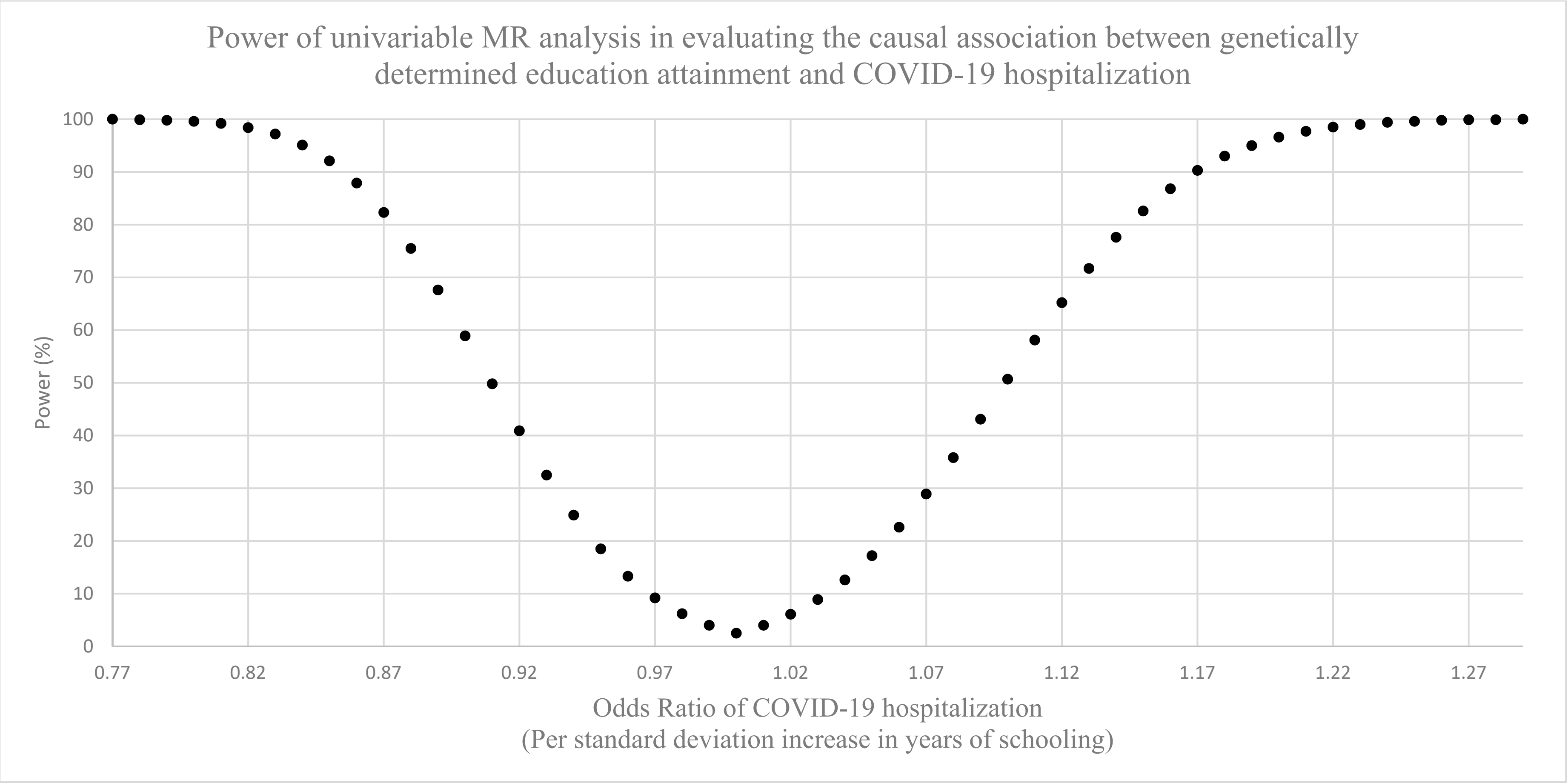

(c) Power of univariable MR analysis in evaluating the causal association between genetically determined education attainment and COVID-19 severity.

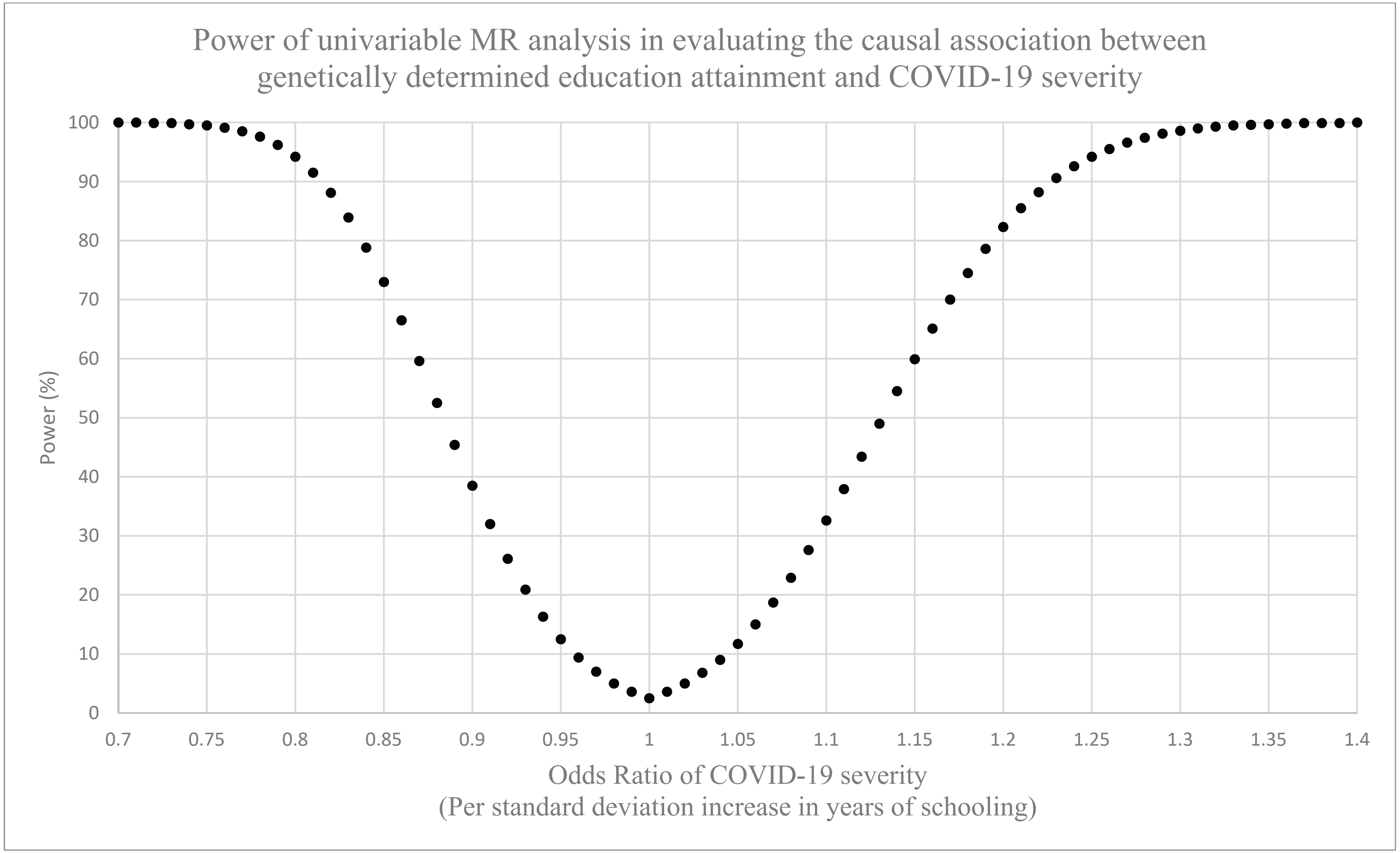

(d) Power of univariable MR analysis in evaluating the causal association between genetically determined intelligence and SARS-CoV-2 infection.

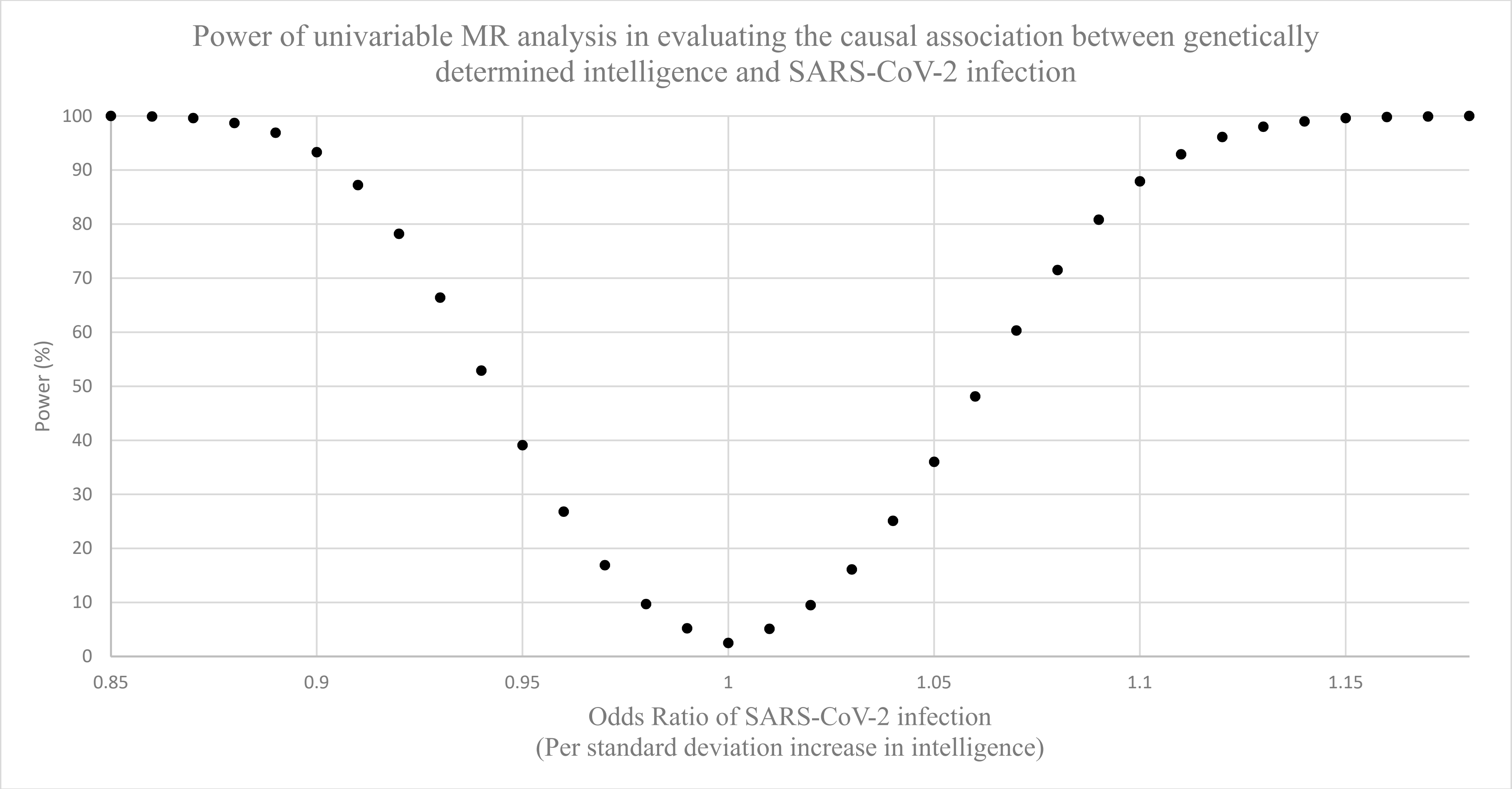

(e) Power of univariable MR analysis in evaluating the causal association between genetically determined intelligence and COVID-19 hospitalization.

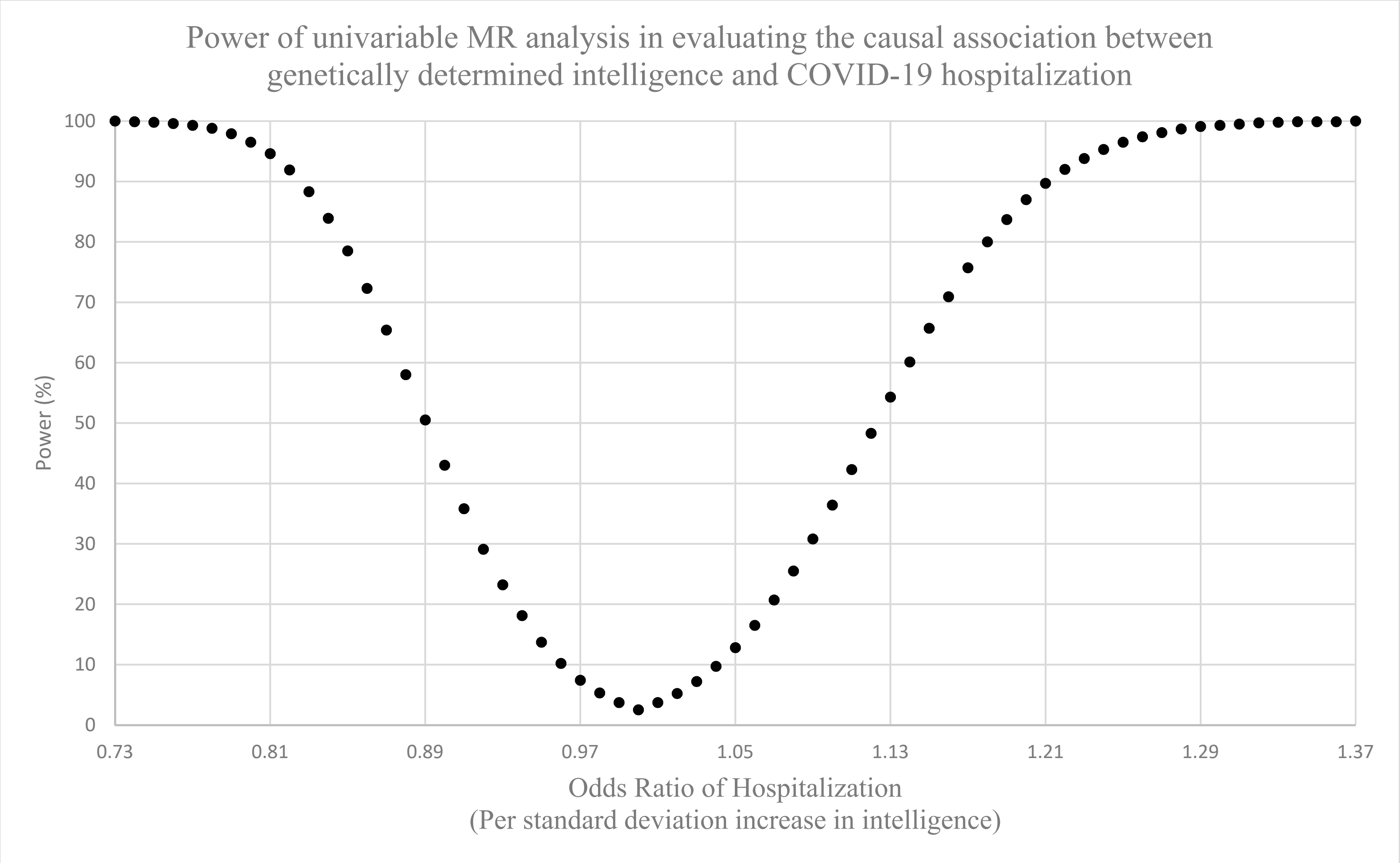

(f) Power of univariable MR analysis in evaluating the causal association between genetically determined intelligence and COVID-19 severity.

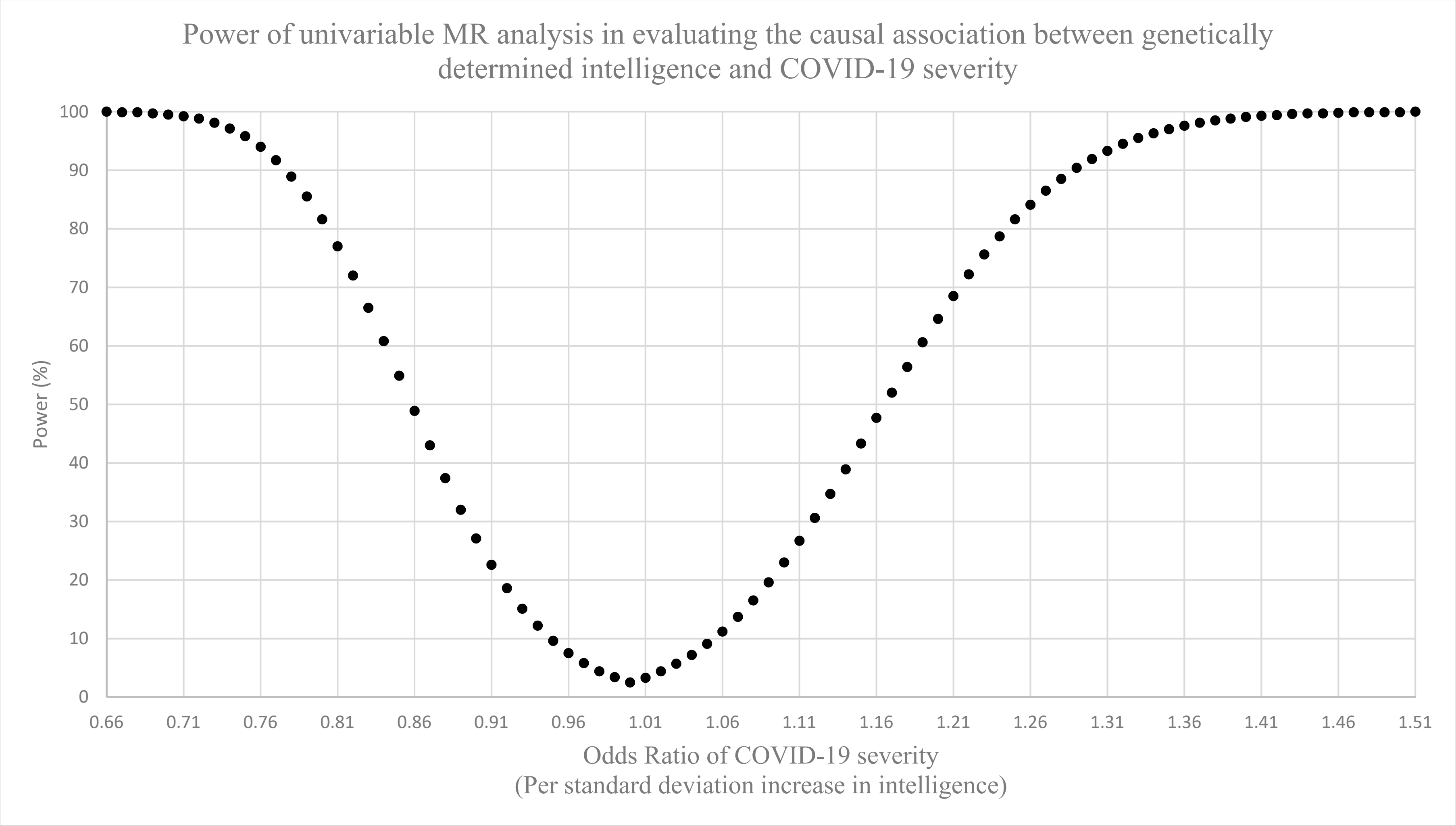

(g) Power of multivariable MR analysis in evaluating the causal association between genetically determined education attainment and COVID-19 hospitalization.

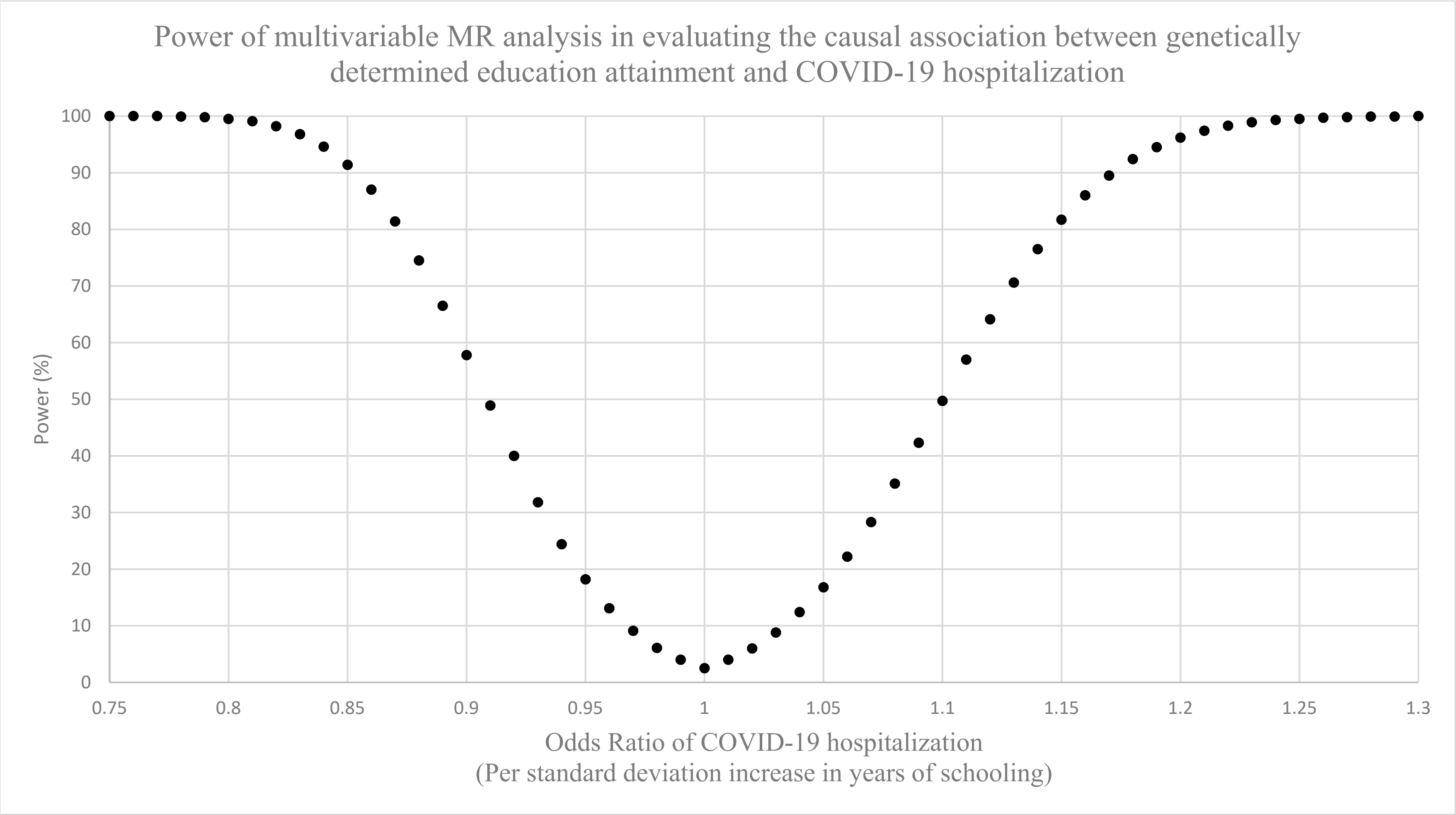

(h) Power of multivariable MR analysis in evaluating the causal association between genetically determined education attainment and COVID-19 severity.

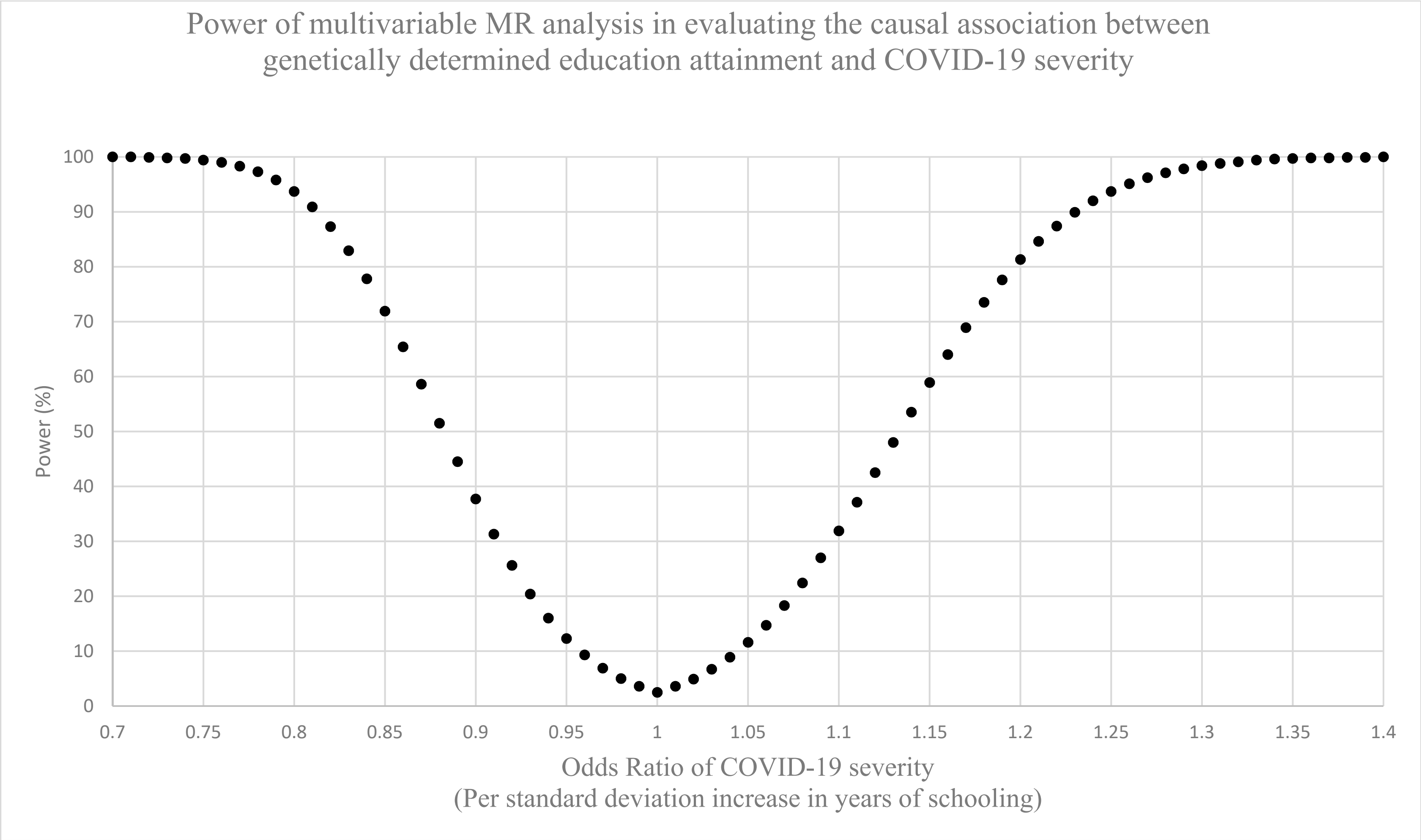

(i) Power of multivariable MR analysis in evaluating the causal association between genetically determined intelligence and COVID-19 hospitalization.

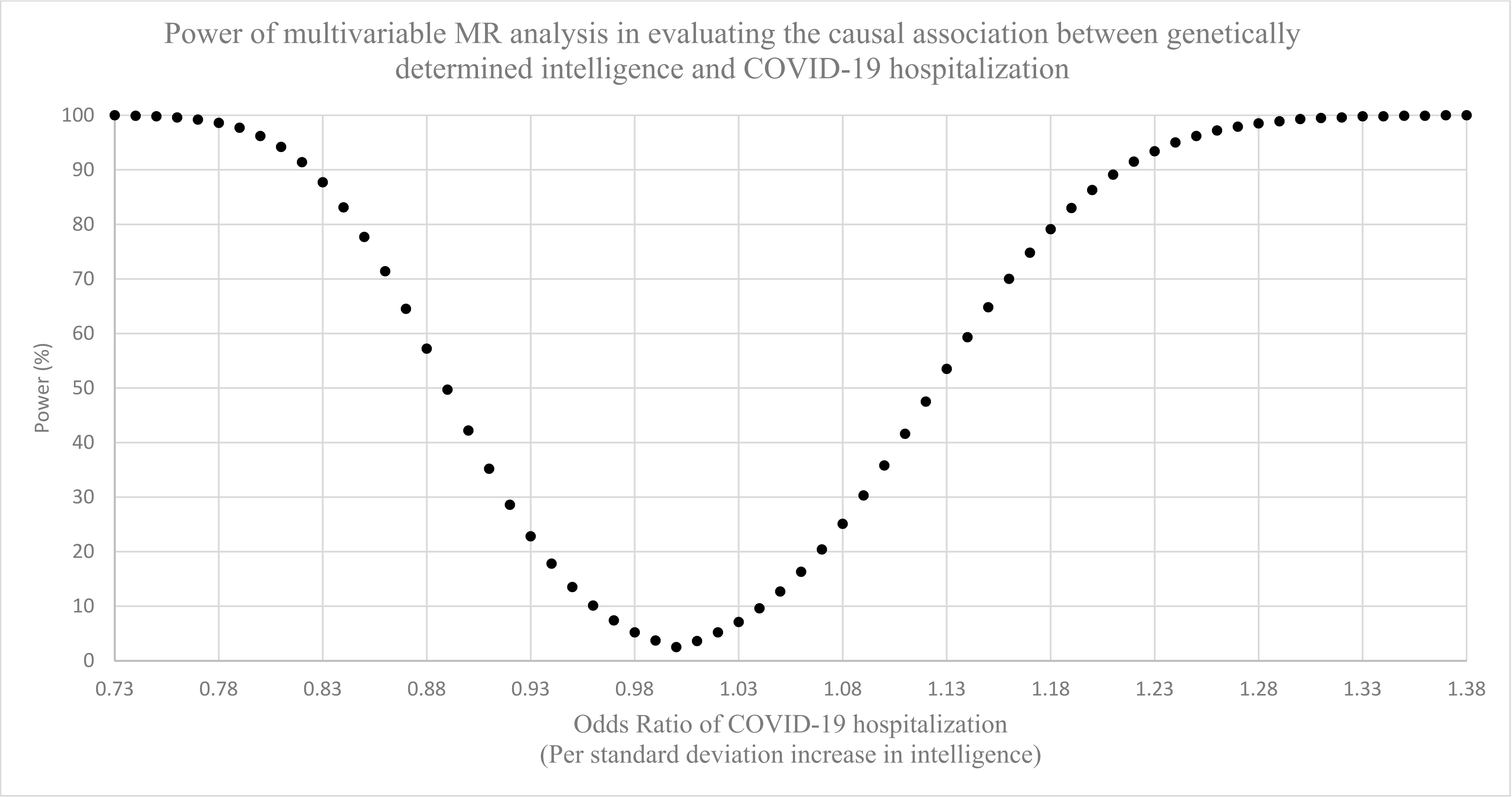

Supplementary References

1. Lee JJ, Wedow R, Okbay A, et al. Gene discovery and polygenic prediction from a genome-wide association study of educational attainment in 1.1 million individuals. Nat Genet **2018**; 50:1112-21.
2. Savage JE, Jansen PR, Stringer S, et al. Genome-wide association meta-analysis in 269,867 individuals identifies new genetic and functional links to intelligence. Nat Genet **2018**; 50:912-9.
3. Bowden J, Spiller W, Del Greco MF, et al. Improving the visualization, interpretation and analysis of two-sample summary data Mendelian randomization via the Radial plot and Radial regression. Int J Epidemiol **2018**; 47:1264-78.
4. Hartwig FP, Davies NM, Hemani G, Davey Smith G. Two-sample Mendelian randomization: avoiding the downsides of a powerful, widely applicable but potentially fallible technique. Int J Epidemiol **2016**; 45:1717-26.
5. Burgess S, Butterworth A, Thompson SG. Mendelian randomization analysis with multiple genetic variants using summarized data. Genet Epidemiol **2013**; 37:658-65.
6. Burgess S, Thompson DJ, Rees JMB, Day FR, Perry JR, Ong KK. Dissecting Causal Pathways Using Mendelian Randomization with Summarized Genetic Data: Application to Age at Menarche and Risk of Breast Cancer. Genetics **2017**; 207:481-7.
7. Bowden J, Davey Smith G, Haycock PC, Burgess S. Consistent Estimation in Mendelian Randomization with Some Invalid Instruments Using a Weighted Median Estimator. Genet Epidemiol **2016**; 40:304-14.
8. Bowden J, Davey Smith G, Burgess S. Mendelian randomization with invalid instruments: effect estimation and bias detection through Egger regression. Int J Epidemiol **2015**; 44:512-25.
9. Burgess S, Foley CN, Allara E, Staley JR, Howson JMM. A robust and efficient method for Mendelian randomization with hundreds of genetic variants. Nat Commun **2020**; 11:376.
10. Slob EAW, Burgess S. A comparison of robust Mendelian randomization methods using summary data. Genet Epidemiol **2020**; 44:313-29.
11. Verbanck M, Chen CY, Neale B, Do R. Detection of widespread horizontal pleiotropy in causal relationships inferred from Mendelian randomization between complex traits and diseases. Nat Genet **2018**; 50:693-8.
12. Burgess S, Thompson SG. Multivariable Mendelian randomization: the use of pleiotropic genetic variants to estimate causal effects. Am J Epidemiol **2015**; 181:251-60.
13. Rees JMB, Wood AM, Burgess S. Extending the MR-Egger method for multivariable Mendelian randomization to correct for both measured and unmeasured pleiotropy. Stat Med **2017**; 36:4705-18.
14. Yavorska OO, Burgess S. MendelianRandomization: an R package for performing Mendelian randomization analyses using summarized data. Int J Epidemiol **2017**; 46:1734-9.
15. Burgess S, Davies NM, Thompson SG. Bias due to participant overlap in two-sample Mendelian randomization. Genet Epidemiol **2016**; 40:597-608.
16. Burgess S. Sample size and power calculations in Mendelian randomization with a single instrumental variable and a binary outcome. Int J Epidemiol **2014**; 43:922-9.
17. Tillmann T, Vaucher J, Okbay A, et al. Education and coronary heart disease: mendelian randomisation study. BMJ **2017**; 358:j3542.
18. Aghili SMM, Ebrahimpur M, Arjmand B, et al. Obesity in COVID-19 era, implications for mechanisms, comorbidities, and prognosis: a review and meta-analysis. Int J Obes (Lond) **2021**; 45:998-1016.
19. Usman MS, Siddiqi TJ, Khan MS, et al. Is there a smoker's paradox in COVID-19? BMJ Evid Based Med **2020**.

20. Fluharty ME, Pinto Pereira SM, Benzeval M, et al. Educational differentials in key domains of physical activity by ethnicity, age and sex: a cross-sectional study of over 40 000 participants in the UK household longitudinal study (2013-2015). BMJ Open **2020**; 10:e033318.
21. Nigro E, Polito R, Alfieri A, et al. Molecular mechanisms involved in the positive effects of physical activity on coping with COVID-19. Eur J Appl Physiol **2020**; 120:2569-82.
22. Landmesser U, Lehmann I, Eils R. Hyperinflammation as underlying mechanism predisposing patients with cardiovascular diseases for severe COVID-19. Eur Heart J **2021**; 42:1720-1.
